# Mental Health and Religiosity in the Sardinian Blue Zone: Life Satisfaction and Optimism for Aging Well

**DOI:** 10.1007/s10943-021-01261-2

**Published:** 2021-04-21

**Authors:** Maria Chiara Fastame, Marilena Ruiu, Ilaria Mulas

**Affiliations:** Department of Pedagogy, Psychology, Philosophy, University of Cagliari, Via Is Mirrionis 1, 09123 Cagliari, Italy

**Keywords:** Life satisfaction, Aging, Optimism, Resilience, Religiosity

## Abstract

This study evaluated the impact of the sociocultural context on dispositional optimism and resilience, life satisfaction, and religiosity in late adulthood. Moreover, the associations between those psychological measures and religiosity were investigated. Ninety-five older individuals recruited in the Sardinian Blue Zone and Cagliari completed a battery of tools assessing cognitive and mental health, and religiosity. Life satisfaction correlated with resilience and religiosity, whereas resilience correlated with optimism. Furthermore, participants of the rural area reported greater optimism and life satisfaction than peers living in the urban area. In conclusion, optimism and hedonic well-being favor optimal aging in the Blue Zone.

## Introduction

The rising prevalence of octogenarians and older individuals requires the implementation of specific interventions aimed at enabling “as many people as possible to be as healthy as possible for as long as possible” (WHO, 2020). In this regard, a body of studies is focused on the individuation of factors promoting successful aging (i.e., an adaptive process reflecting the interplay between gains and losses) that encompasses compensatory efforts to reduce the aging effects through a set of selected behaviors aimed at empowering the residual domains of functioning (Baltes & Baltes, 1990).

### Dispositional Optimism, Life Satisfaction, and Resilience to Promote Successful Aging

There is evidence that successful aging is favored by dispositional optimism, that is to say, a personality trait reflecting the attitude that beneficial events will happen or that the future will be favorable thanks to the control of crucial factors (Scheier & Carver, 1985). In this regard, it has been found that higher levels of optimism occur with healthier physical habits (e.g., regular physical activity, healthy diet, greater therapeutic adherence), which, in turn, reduce the risk of chronic pathologies and increase life expectancy (Boehm et al., 2018; Progovac et al., 2017; Steptoe et al., 2006). Following this, a longitudinal study conducted with physically and cognitively intact Americans that at baseline were over 50 years old documented that after controlling for sociodemographic factors and depression, the most optimistic participants (i.e., top quartile) had a 24% increased likelihood of becoming healthy older individuals (i.e., those displaying reduced risk of cognitive and physical deterioration) over 6–8 years of follow-up (Kim et al., 2019). Consistently, according to Lee et al. (2019), higher optimism is linked to higher odds of achieving exceptional longevity (i.e., being 85 years old and over) both in men and in women.

Additionally, Cosco et al. (2014) suggest that among others, life satisfaction and personal resources such as resilience are important outcomes associated with successful aging. Life satisfaction refers to a facet of hedonic well-being encompassing a cognitive-judgmental process driving people in comparing their actual level of pleasure and satisfaction with their standard criteria (Diener et al., 1985). Moreover, dispositional resilience concerns “the capacity to maintain or regain well-being in the face of adversity” (Ryff, 2014, p. 10) and facing challenges in life. Extending this, according to Sisto et al. (2019), resilience encompasses the attitude to persist in the orientation to pursue existential long-term purposes according to one’s life commitments (e.g., vocational, social, professional).

There is an increasing body of knowledge about the strong positive association in the late adulthood between life satisfaction and optimism (Dumitrache et al., 2019; Ju et al., 2013), which, in turn, boosts resilience (Baldwin et al., 2011). Overall, resilience is an adaptive and protective resource promoting different dimensions of life quality, such as psychological well-being (e.g., life satisfaction) and physical health (Nalin & Franca, 2015). Therefore, according to Dumitrache et al. (2015), more optimistic older individuals tend to adapt better to the losses characterizing aging, that is, these older people perform only tasks that they can still carry out, in order to maintain adequate levels of life satisfaction. Furthermore, Zeng and Shen (2010) documented the significant association between resilience and exceptional longevity, showing that the most resilient nonagenarians were 43% more likely to become centenarians.

### Religiosity for Aging Well

A further protective factor for aging well is religiosity, since older individuals attributing much value to religion in their life also report more life satisfaction (Fastame et al., 2017; Krause, 2003). Extending this, a related body of studies conducted in a Christian context documented the protective effects of meditative prayer (i.e., a set of individual- and focus-group prayers based on the contemplation of spiritual contents and the relationship of God with human beings considered a form of transcendental meditation) in preventing burnout and enriching psychological well-being in a group of Italian teachers (Chirico et al., 2020). Further studies revealed the effectiveness of spiritual interventions for the enhancement of mental health (e.g., optimism, life satisfaction) and the reduction of negative emotional outcomes (i.e., anxiety, depression) in late adulthood (e.g., Ai et al., 2002; Rajagopal et al., 2002). Indeed, researchers acknowledged that spiritual behaviors (e.g., church attendance, individual prayer) favor the adaptation of older individuals to the physically and psychologically stressful challenges (e.g., onset of chronical disorders threatening the quality of life, loss of the spouse) that they must face, especially in the last decades of life when people also use religiosity as an effective coping strategy to prepare themselves for the end of life (Bishop, 2011).

### Well-Being in the Sardinian Blue Zone

So far, to our knowledge, no studies have been conducted to examine whether optimism, life satisfaction, resilience, and religiosity are particularly effective in protecting subjective mental health of longevous people living in the Blue Zones. These are geographic spots of exceptional longevity, being characterized by the highest prevalence of centenarians. The Blue Zones are five rural spots, and they are located in Greece (i.e., Ikaria), Italy (in the central-eastern area of Sardinia), Japan (i.e., Okinawa), Costa Rica (i.e., Nicoya), and the USA (i.e., Loma Linda), respectively (Poulain et al., 2013).

The Italian Blue Zone is a longevity spot located in the mountainous area of Sardinia, an island in the Mediterranean Sea where a very simple lifestyle prevails. In the Sardinian Blue Zone, a high proportion of shepherds preserving their cultural traditions and being more resistant to change live and the highest gender-balanced presence of centenarians in the world has been experimentally validated (Pes et al., 2015; Poulain et al., 2004). The successful aging of Sardinians living in the Blue Zone is considered multicausal, that is, it seems to be determined by lifestyle (e.g., engagement in outdoor physically and socioculturally oriented leisure activities), a plant-based diet associated with moderate consumption of meat and dairy products, genetic, environmental (e.g., the higher terrain steepness of the territory induces higher physical and cardiovascular activities), physiological metabolic, sociocultural, and psychological factors (e.g., Fastame et al., 2015; Manca et al., 2021; Nieddu et al., 2020; Pes et al., 2013). Regarding the psychosocial dimension of the successful aging in Sardinia, a strand of research has documented that perceived mental health (e.g., negative affect, psychological well-being) of community-based and cognitively intact older individuals of the Sardinian Blue Zone is more preserved compared to that of their peers living in rural areas of Northern Italy or in the Sardinian urban area (for a review, see Hitchcott et al., 2018). According to Fastame et al. (2014) compared to their peers recruited in the rural areas of Northern Italy, in addition to a more preserved mental health, the older inhabitants of the Sardinian Blue Zone also reported being more respected or as respected as in the past by the young people, and that their villages were the best places to grow old. In contrast, the older people recruited in Northern Italy reported to be not respected and that successful aging could be possible but elsewhere. Therefore, Fastame et al. (2014) concluded that the prevalence of the collectivistic culture in the Sardinian context where the older people are considered a resource for their community represents a protective factor for aging well. Extending this, some ethnographic investigations documented that in the mountainous area of Sardinia the religious practices (which are mainly performed by middle-aged and older Catholic women) are collective historical performances evoking local rituals (e.g., women in local costumes singing the rosary in the local dialect), which reinforce the social identity and the engagement of the whole local community in many religious events (Heatherington, 1999). In this perspective, in the Sardinian context the religious events do have a spiritual, social, cultural, folkloristic, and historical connotation in which congregations of believers are actively involved in the organization.

Moreover, Fastame et al. (2018) showed that optimal regulation, which is a facet of resilience promoting effective management of negative emotionality, significantly predicted negative affect and psychological well-being of older people of the Sardinian Blue Zone. Extending this, the first longitudinal study conducted in the Sardinian Blue Zone recently documented that compared to national parameters, the older inhabitants of the longevity area displayed preserved motor efficiency, higher perceived psychological well-being, and fewer depressive symptoms even 2 years after the first assessment (Fastame, Mulas & Pau, 2020). However, thus far, no studies have been conducted to explore extensively whether the protective factors promoting successful aging are more preserved in people living in areas of exceptional longevity than in those living in urban areas. Indeed, to our knowledge, only one study highlighted that older people living in the Sardinian Blue Zone reported fewer depressive signs than peers living in Sassari (a Sardinian town located in the northwest part of the island) and in Lombardy (Fastame et al., 2015). In contrast, so far, no studies have examined the impact of religiosity on successful aging. Indeed, the only study conducted with Sardinian older participants documented moderate and positive relationships among religiosity and perceived psychological well-being in late adulthood (Fastame et al., 2017).

### The Study

This study is mainly intended to explore whether older individuals (i.e., 70 years old and older) living in the Sardinian Blue Zone reported better perceived mental health (i.e., optimism, life satisfaction, resilience) and higher religiosity than peers residing in a Sardinian urban area. A further aim was to examine the nature of the associations between optimism, resilience, life satisfaction, and religiosity in the late adult lifespan. To pursue the aims of this investigation, the current data were partially collected in a village located in the Sardinian Blue Zone, that, unlike the other Blue Zones, is characterized by a similar prevalence of male centenarians to that of women centenarians (Poulain et al., 2004).

Following Fastame et al. (2015), it was hypothesized that life satisfaction was greater in older people of the Blue Zone than in participants living in the urban area. Furthermore, if resilience and optimism concur to promote successful aging and longevity (Cosco et al., 2014; Zeng & Shen, 2010), these resources were expected to be more developed in older people of the Sardinian Blue Zone. Moreover, females were expected to report higher religiosity than older men (Leondari & Gialamas, 2009). Additionally, significant associations were hypothesized in older individuals between life satisfaction and optimism (Dumitrache et al., 2015, 2019; Ju et al., 2013) and between the former and religiosity (Bishop, 2011; Chirico et al., 2020; Fastame et al., 2017; Krause, 2003). Finally, life satisfaction and optimism were expected to be related to resilience (Baldwin et al., 2011; Nalin & Franca, 2015).

## Method

### Participants

Ninety-five cognitively healthy older individuals (mean age = 79.6 years, SD = 6.3, age range = 70–94 years) took part in the study. Specifically, 48 participants (20 males) were recruited in the urban area of Cagliari (i.e., the main Sardinian town and political center of the island), whereas 47 respondents (25 males) lived in an agro-pastoral village of the Sardinian Blue Zone.

The recruitment of the participants was conducted through personal contacts with the local social agencies for older people and applying the snowball sampling procedure. All the respondents were volunteers, and they did not receive any incentives or compensation to take part in the study.

To be enrolled in the study, the following inclusion criteria were used: 1) to be born in the metropolitan area of Cagliari or the Sardinian Blue Zone; 2) to be descendant of families living in those areas for at least two previous generations; 3) to be community-based (i.e., not residing in a nursing home for older people); 4) to be cognitively healthy, that is, a score ≥ 24/30 using the Mini-Mental State Examination test (MMSE, Folstein et al., 1975) had to be reported; 5) to be at least 70 years old. Table 1 summarizes the characteristics of the sample.

**Table 1:** Sociodemographic information and global cognitive efficiency (i.e., MMSE) of the participants of the study enrolled in the urban area of Cagliari and the Sardinian Blue Zone

Variable	Cagliari	Sardinian Blue Zone	*t*	*df*	*p*
*n*	48	47			
Gender					
Males	20	25			
Females	28	22			
Marital status					
Married	29	25			
Single	19	22			
Age (years)	*M* = 79.2 (SD = 5.6)	*M* = 80 (SD = 7.3)	− .59	93	.56
MMSE	*M* = 27.4 (SD = 1.33)	*M* = 27.3 (SD = 1.56)	.39	93	.70
Education					
Low (0–8 years)	18	30			
High (> 8 years)	30	17			

### Materials

Participants were invited to complete a battery of tasks including:

A sociodemographic interview that was used to record information about lifestyle (e.g., habit of smoking, time spent for leisure activities) and sociodemographic information (e.g., marital status, gender, age, educational attainment) of the participants.

The MMSE (Folstein et al., 1975) is a screening test that was used to assess global cognitive efficiency. This tool encompasses a set of items assessing the efficiency of distinct cognitive processes (e.g., short- and long-term memory, visuo-motor coordination). Following Magni et al. (1996), the total score was adjusted for educational attainment and age. A score < 24 was used to exclude participants with suspected signs of cognitive deterioration.

The Life Orientation Test-Revised (LOT-R, Scheier et al., 1994; Italian version, Anolli, 2005) was used to assess dispositional optimism. This tool is a self-reported questionnaire encompassing six items measuring pessimistic and optimistic attitudes. The participants were asked to express their agreement with each statement using a Likert scale ranging from 1 (strongly disagree) to 5 (strongly agree). After reversing the score of the pessimism-related items, a total score was computed (maximum score = 30).

The SODdisfazione dell'Anziano (SODA) Questionnaire (Fastame, Penna & Hitchcott, 2020) is a self-reported tool including 14 items designed to assess life satisfaction during the past week. Participants were invited to express their agreement with each statement along a Likert scale ranging from 0 (i.e., completely unsatisfied) to 10 (i.e., completely satisfied). The total score was computed (maximum score = 140).

The Dispositional Resilience Scale (DRS-15, Bartone, 2007; Italian version, Picardi et al., 2012) encompassing 15 items was used to assess a personal stress resilience resource, namely the psychological hardiness, that is used to construct meaning even when stressful events occur. For each statement, the participant had to express his/her agreement on a 4-point scale ranging from 0 (not at all true) to 3 (completely true). A total score was calculated (maximum total score = 60).

The Religiosity index (Fastame et al., 2017) was used as a subjective measure of the importance of religion in one’s life. Each respondent had to assess his/her degree of religiosity on a Likert scale ranging from 0 (lack of importance attributed to the religion) to 10 (maximum importance attributed to the religion).

### Procedure

After that written informed consent was provided by each respondent, he/she was individually tested in a quiet room of his/her home. Once that the global cognitive efficiency was tested via the MMSE, if no signs of cognitive deteriorations were displayed (i.e., MMSE score < 24), the sociodemographic interview was conducted, and thereafter, the presentation order of the other questionnaires was counterbalanced across the participants according to the Latin square procedure. In order to avoid the fatigue effect and missing data, the second and third authors read aloud each item of the questionnaires/test and recorded the responses provided by the participants. Overall, the experimental session lasted approximately 50 min.

### Data Analysis

The statistical analyses were performed using version 1.2 of the Jamovi open-source package (Jamovi Project, 2020). Statistical significance was set to *p* values < 0.05. Descriptive statistics were computed to investigate the sociodemographic characteristics of the participants. Then, a series of analyses of the covariance (ANCOVAs) were carried out, to explore the effect of the residential area on the mental health indexes, whilst controlling for the age effect. Besides, a further ANCOVA was performed to examine the effect of the sociocultural context and gender on the religiosity index, using age as a covariate. Finally, Pearson’s correlation coefficients (*r*) were calculated to examine the nature of the relationships among religiosity, life satisfaction, optimism, and resilience.

## Results

First, some preliminary descriptive analyses were performed. No significant differences in terms of age (*t*(93) = − 0.591, *p* = 0.556) and MMSE scores (*t*(93) = 0.388, *p* = 0.70) were reported by the respondents living in in the Sardinian Blue Zone and Cagliari. Moreover, gender (*χ*^2^ = 1.27, *df* = 1, *p* = 0.26) and marital status (*χ*^2^ = 0.505, *df* = 1, *p* = 0.48) were counterbalanced across the areas of residency (i.e., urban vs. Blue Zone). In contrast, as expected, participants living in the urban area were more educated (*M* years of formal schooling = 9.5, SD = 4.7 years) than older individuals of the Sardinian Blue Zone (*M* = 6.5 years, SD = 3.6 years) (*t*(93) = 3.5, *p* < 0.001).

Then, three separate ANCOVAs were conducted to explore the impact of the residential area (urban vs. Blue Zone) on optimism, life satisfaction, and resilience, using age as a covariate. The main effect of residential area was significant in LOT-R [*F*(1,92) = 5.387, *p* = 0.022, *ηp*^2^ = 0.05] and SODA [*F*(1,79) = 4.181, *p* = 0.044, *ηp*^2^ = 0.05] conditions, but not in the resilience one [*F*(1,92) = 0.509, *p* = 0.477]. Thus, participants recruited in the Blue Zone reported better optimism (*M* = 24.34, SD = 4.64) and greater life satisfaction (*M* = 115.38, SD = 14.51) than older individuals living in the town of Cagliari (mean SODA score = 108.21, SD = 17.16 and mean LOT-R score = 22.27, SD = 3.92). Moreover, the effect of age was not significant in any condition [*F*(1,92) = 0.027, *p* = 0.869 for LOT-R, *F*(1,79) = 1.697, *p* = 0.197 for SODA and *F*(1,92) = 1.411, *p* = 0.238 for the resilience score].

A further ANCOVA was conducted to investigate the impact of residential area and gender on religiosity, controlling for the age effect. It was found only the main effect of gender [*F*(1,90) = 6.038, *p* = 0.016, ηp^2^ = 0.06], whereas the main effects of residential area [*F*(1,90) = 0.480, *p* = 0.49] and age [*F*(1,90) = 0.314, *p* = 0.577] were not significant. Table 2 illustrates the mean scores in each of the above-mentioned measures being reported by the participants living in the urban area and the Blue Zone.

**Table 2:** Optimism (i.e., LOT-R), life satisfaction (i.e., SODA), resilience (i.e., DRS-15), and religiosity scores reported by the participants living in the urban area of Cagliari and the Sardinian Blue Zone, respectively

Measure	Cagliari	Sardinian Blue Zone
LOT-R	*M* = 22.27 (SD = 3.92)	*M* = 24.34 (SD = 4.64)
SODA	*M* = 108.21 (SD = 17.16)	*M* = 115.38 (SD = 14.51)
DRS-15	*M* = 28.35 (SD = 4.57)	*M* = 27.57 (SD = 5.08)
Religiosity		
Total	*M* = 7.87 (SD = 2.99)	*M* = 8.19 (SD = 2.89)
Men	*M* = 7.7 (SD = 2.64)	*M* = 6.96 (SD = 3.06)
Women	*M* = 8 (SD = 3.25)	*M* = 8.19 (SD = 2.88)

Standard deviations are illustrated in parentheses

Finally, Pearson product moment correlations were carried out among life satisfaction (i.e., SODA), optimism (i.e., LOT-R), resilience (i.e., DRS-15) and religiosity, respectively. Significant associations were found between SODA and DRS-15 (*r* = 0.274, *p* = 0.013) and religiosity (*r* = 0.591, *p* < 0.001), respectively. Additionally, DRS-15 and LOT-R were significantly associated (*r* = 0.204, *p* 0.048). In contrast, the associations between LOT-R and SODA (*r* = 0.18, *p* = 0.107), between religiosity and LOT-R (*r* = 0.024. *p* = 0.82) and between religiosity and resilience (*r* = − 0.047, *p* = 65) were not statistically significant.

## Discussion and Conclusions

The purpose of this investigation was twofold. The primary goal was to examine the impact of the sociocultural context on psychological markers of successful aging (i.e., life satisfaction, optimism, resilience, and religiosity), using age as covariate. Moreover, the nature of the associations between dispositional optimism and resilience, life satisfaction, and religiosity was also analyzed.

Current findings extend previous evidence highlighting the protective factors promoting optimal aging. Specifically, extending evidence documented by Fastame et al. (2015), older people of the Sardinian Blue Zone reported greater dispositional optimism and life satisfaction than participants recruited in the city of Cagliari. Specifically, using the Italian normative data proposed by Anolli (2005), optimism scores of the participants recruited in the Sardinian Blue Zone were very high, since they were reported to be beyond the 80^th^ percentile, whilst the measures of the participants of the urban area fell around the 50 ^th^ percentile. Moreover, although the respondents of the rural and urban areas reported very high life satisfaction indexes (Fastame, Penna & Hitchcott, 2020), it must be noticed that those of the Sardinian Blue Zone reported greater hedonic well-being.

However, current results are only partially consistent with those documented by Cosco et al. (2014) and Zeng and Shen (2010), since the sociocultural context does not impact resilience, that is, participants of the rural and urban areas reported mean scores similar to those reported by younger adults (Picardi et al., 2012). Additionally, both the groups (i.e., those living in the rural area and those living in Cagliari) exhibited high levels of religiosity, but, as expected (Leondari & Gialamas, 2009), women reported higher religiosity than males. Overall, these outcomes suggest that religiosity is a protective factor for optimal aging, that is, religiosity is a coping strategy that is particularly useful for the older women, who are more prone to experiencing more depressive symptoms and anxiety (for a review, see Djernes, 2006).

Further, when the nature of the associations between the dispositional resources, hedonic well-being and religiosity was evaluated, our hypotheses were partially confirmed. Specifically, in line with previous studies (Fastame et al., 2017; Krause, 2003), using the effect size parameters suggested by Cohen (1988), a large correlation (*r* > 0.05) was found between life satisfaction and religiosity, and the former was also moderately associated with resilience (Nalin & Franca, 2015). Similarly, as expected (Baldwin et al., 2011), significant moderate associations were also found between dispositional resilience and optimism. Therefore, as already suggested by Sisto et al. (2019), resilience is a dynamic and adaptive psychological resource for the maintenance of one’s orientation toward existential (e.g., vocational) purposes and to being open to change despite the occurrence of adversities and stressful events, that concurs for the promotion of mental well-being in the late adult life span. However, contrary to our expectations, no significant correlations were found between optimism and life satisfaction and the former and religiosity, respectively (Dumitrache et al., 2015, 2019; Ju et al., 2013). Overall, these findings allow us to speculate that older people reporting greater religiosity use it as coping strategy to deal with stressful events; therefore, they appear more resilient and are more optimistic toward their existential purposes in life. All these resources dynamically and adaptively concur in promoting mental health, since more resilient and religious people also exhibited more hedonic well-being.

Altogether, embracing an applied approach, current outcomes suggest that in order to promote optimal aging (e.g., maintenance of healthy habits, cognitive and mental health), the implementation of interventions fostering psychological well-being and boosting dispositional optimism are required (Cosco et al., 2014; WHO, 2020). Concerning this, numerous studies highlighted the effectiveness of spirituality and religious activity as a coping strategy for the empowerment of mental health (e.g., life satisfaction, happiness, optimism) and the management of stress-related events across the adult life span (e.g., Chirico, 2017; Frankel & Hewitt, 1994; Lawler-Row & Elliott, 2009). There is evidence that interventions based on the use of meditational techniques and prayer can be very effective in reducing emotional exhaustion and enhancing mental health (e.g., reducing depressive symptoms, anxiety) in adults (Chirico et al., 2020), especially in older individuals who have to deal with different types of stressful events (e.g., loss of relatives, occurrence of chronic diseases) threatening their mental health. In this regard, spirituality-based interventions are effective in reducing depression and anxiety and empowering optimism among older individuals (Rajagopal et al., 2002), even among those exhibiting cardiovascular diseases (Ai et al., 2002).

Nonetheless, some methodological limits of this study must be pointed out. The limited battery of tasks and the sample size, and the recruitment of only community-based participants, suggest caution in generalizing these findings. It is desirable that future research will replicate this investigation, possibly longitudinally, both in further Blue Zones and with institutionalized participants. Finally, it is desirable that this study will be replicated with older individuals exhibiting a few signs of cognitive decline (i.e., mild cognitive decline), in order to evaluate the impact of cognitive deterioration on aging of people living in an area of exceptional longevity.

## Data Availability

The data that support the findings of this study are not publicly available due to privacy or ethical restrictions.
