# Correction to: Mental Health and Religiosity in the Sardinian Blue Zone: Life Satisfaction and Optimism for Aging Well

**DOI:** 10.1007/s10943-021-01288-5

**Published:** 2021-05-19

**Authors:** Maria Chiara Fastame, Marilena Ruiu, Ilaria Mulas

**Affiliations:** Department of Pedagogy, Psychology, Philosophy, University of Cagliari, Via Is Mirrionis 1, 09123 Cagliari, Italy

## Correction to: Journal of Religion and Health https://doi.org/10.1007/s10943-021-01261-2

In the original publication of the article, the author noticed the errors in the reference citation.

The corrected text citations are given below:

In section, “Well‑Being in the Sardinian Blue Zone”, last paragraph, the second sentence should read, “Extending this, the first longitudinal study conducted in the Sardinian Blue Zone recently documented that compared to national parameters, the older inhabitants of the longevity area displayed preserved motor efficiency, higher perceived psychological well-being, and fewer depressive symptoms even 2 years after the first assessment (Fastame, Mulas & Pau, 2020).”

In Materials section, fourth paragraph, the first sentence should read,” The SODdisfazione dell’Anziano (SODA) Questionnaire (Fastame, Penna & Hitchcott, 2020) is a self-reported tool including 14 items designed to assess life satisfaction during the past week.”

In Discussion and Conclusions, second paragraph, the last sentence should read, “Moreover, although the respondents of the rural and urban areas reported very high life satisfaction indexes (Fastame, Penna & Hitchcott, 2020), it must be noticed that those of the Sardinian Blue Zone reported greater hedonic well-being”.

The original article has been corrected.
